# Linear Processes in Stochastic Population Dynamics: Theory and Application to Insect Development

**DOI:** 10.1155/2014/873624

**Published:** 2014-02-17

**Authors:** Hernán G. Solari, Mario A. Natiello

**Affiliations:** ^1^Departamento de Física, FCEN-UBA and IFIBA-CONICET, C1428EGA Buenos Aires, Argentina; ^2^Centre for Mathematical Sciences, Lund University, P.O. Box 118, 221 00 Lund, Sweden

## Abstract

We consider stochastic population processes (Markov jump processes) that develop as a consequence of the occurrence of random events at random time intervals. The population is divided into subpopulations or compartments. The events occur at rates that depend linearly on the number of individuals in the different described compartments. The dynamics is presented in terms of Kolmogorov Forward Equation in the space of events and projected onto the space of populations when needed. The general properties of the problem are discussed. Solutions are obtained using a revised version of the Method of Characteristics. After a few examples of exact solutions we systematically develop short-time approximations to the problem. While the lowest order approximation matches the Poisson and multinomial heuristics previously proposed, higher-order approximations are completely new. Further, we analyse a model for insect development as a sequence of *E* developmental stages regulated by rates that are linear in the implied subpopulations. Transition to the next stage competes with death at all times. The process ends at a predetermined stage, for example, pupation or adult emergence. In its simpler version all the stages are distributed with the same characteristic time.

## 1. Introduction

Stochastic population dynamics deals with the (stochastic) time evolution of groups of similar individuals (called populations or subpopulations) immersed in a habitat. The goal is to describe the evolution of population numbers and the number of events associated with changes in the populations.

Most of the attention has been paied in the past to the populations, considering events only by their extrinsic value as jumps in populations. However, this perspective must be revised. In any study of vector-transmitted diseases, such as in [1, 2], the most relevant statistics corresponds to the interactions between hosts and vectors. Such interactions are associated with events in the life cycle of the vector. In the case of arthropods transmitting diseases to mammals, blood feeding to complete oogenesis is the opportunity to transmit pathogens. In other problems, the development of insects is experimentally described by the sequences of transformations in their life cycle (such as moulting) [3–5]: each transformation must be considered an event. To our knowledge, explicit use of the event structure to study population changes in Markov Jump processes has been introduced in [6], along with a short-time approximation based on self-consistent Poisson processes.

The present research originates in our efforts to model vector-transmitted diseases, such as Dengue, starting from a description of the life cycle of *Aedes aegypti*, the vector [7–9]. In modeling insect development in an aquatic phase (such as the case of *Aedes aegypti* and *Drosophila melanogaster*) a crucial point is to represent accurately the statistics of the duration of the aquatic phase (also known as the time of emergence statistics) as it determines the response of the population to climatic events such as rains [10]. A deterministic model for development was proposed in [11] as a sequential process with associated linear rates. The final sections of this paper describe a stochastic development model.

While [6] deals with the stochastic approximation of general problems (achieving a method with error *o*(*t* ^3/2^)), this paper specifically addresses the problem of formulating and solving population dynamics problems in event space with *linear* rates, that is, rates that depend linearly on the populations (in the chemical literature they are known as first-order reactions [12]). We provide both exact solutions and general approximation techniques with error *o*(*t* ^*n*^). Results from [6] for the linear case are a modified version of one of the approximation schemes in this paper (Poisson approximation, Section 5). The linear problem is mathematically simpler than the general case and a few general results can be established before resorting to approximations.

Linear processes are an important part of most biological dynamical descriptions. Using an analogy from classical population dynamics, linear (Malthusian) population growth [13] describes the evolution of a population at low densities (e.g., where the local environment is essentially unaffected by the population growth and the individuals do not interfere with each other). More generally, processes that consider only individuals in a somewhat isolated basis can be satisfactorily described with linear rates. However, whether to use linear descriptions or not is ultimately determined by the nature of the process (or subprocess). For example, finite environments will sooner or later force individuals to interact in growing populations and nonlinear rates become necessary such as in Verhulst's description of population growth [14].

Perhaps the oldest application of linear processes to population dynamics regards *birth* and *death* processes. Historically, according to Kendall [15], the first to propose birth and death equations in population dynamics was Furry [16], for a system of physical origin. Currently, such an approach is being used in several sciences: linear stochastic processes have been proposed for the development of tumors [17, 18]; drug delivery and cell replication are also described in these terms [19]. Concerning the tumors, the model proposed in [17, 18] is based on linear individual processes, a detailed treatment of which would require a paper of its own. The accumulation of individual processes is finally approximated with a “tunnelling process” corresponding to a maturation cascade that can be easily described with the tools of this paper (see (71)). Concerning the question of drug delivery, we will come back in detail to [19] in Section 7.

A substantial use of population dynamics driven by jump processes has been done also in chemistry, particularly after the rediscovery by Gillespie [20] of Kendall's simulation method [21, 22]. Poisson [23, 24], binomial [25, 26], and multinomial [27] heuristics for the short-time integrals have been proposed. There is, however, an important difference between chemistry and biology concerning the issues of this paper. On the one hand, linear rates are seldom the case in chemical reactions except perhaps in the case of irreversible decompositions or isomeric recombination. Attention to linear rates in chemistry is relatively recent [12] when compared to over 200 years of Malthusian population growth. On the other hand, detailed event statistics does not appear to be fundamental either: highly diluted chemical reactions on a test tube may easily generate 10^14^ events of each type. Consequently, attention to event based descriptions appears to be absent from the chemistry literature. This might, however, change in the future, since the technical possibilities given by for example, “in-cell chemistry” and “nanochemistry” allow dealing with a lower number of events where tiny differences in event count could be relevant for the general outcome.

In the mathematical literature attention to jump processes starts with Kolmogorov's foundational work [28] and its further elaboration by Feller [29]. A substantial effort to relate the stochastic description to deterministic equations was performed by Kurtz [30–34]. However, attention has always focused on the dynamics in population space, rather than event space.

This work focuses on the event statistics associated with *linear* processes. Even for cases where the detailed event statistics is not of central interest, such an approach offers an alternative to the more traditional formulation in population space.

Further, we discuss in detail the stochastic version of a linear developmental model. Specifically, we deal with a subprocess in the life cycle of insects that admits both a linear description and an exact solution, namely, the development of immature stages, which is a highly individual subprocess. From egg form to adult form, the development of insects goes through several transformations at different levels. For example, at the most visible level, insects undergoing complete metamorphosis evolve in the sequence egg, larva, pupa, and adult. Further, different substages can be recognised by inspection in the development of larvae (instars) and even more stages when observed by other methods [35]. The immature individual may die along the process or otherwise complete it and exit as adult.

In Section 2 we formulate the dynamical population problem in event space. In Section 2.1 we present the Kolmogorov Forward Equations (for Markov jump processes) for the most general linear systems of population dynamics in the form appropriated to probabilities and generating functions. The general solution of these equations will be written using the Theorem of the Characteristics, reformulated with respect to the standard geometrical presentation [36] into a dynamical presentation. We will analyse the general structure of the equations and in particular their first integrals of motion and the projection onto population space (Sections 2 and 3).  Section 4 contains two classical examples. In Section 5, Poisson and multinomial approximations (both with error *o*(*t*)) are derived and a higher-order approximation with error *o*(*t* ^*n*^) (with *n* as an arbitrary positive integer) is elaborated. The larval development model is developed in Sections 6 and 7, treating the two general cases. We also revisit in Section 7 the question of drug delivery introduced in [19], in terms of the present approach. Concluding remarks are left for the final Section.

## 2. Mathematical Formulation

The main tools of stochastic population dynamics are a list of (nonnegative, integer) populations *X*_*j*_, where *j* = 1 ⋯ *N* a list of events *α* = 1 ⋯ *E* an (integer) *incidence matrix δ*_*j*_^*α*^ describing how event *α* modifies population *j*, and a list of transition rates *W*_*α*_(*X*_1_,…, *X*_*n*_, *t*) describing the probability rate per unit time of occurrence of each event.

An important feature of the present approach is that the dynamical description will be handled in event-space. In this sense, the “state” of the system is given by the array **n**(*s*, *t*) = (*n*_1_,…,*n*_*E*_)_*t*_ indicating how may events of each class have occurred from time *s* up to time *t*. Hence, *event-space* is, in this setup, a nonnegative integer lattice of dimension *E*. The connection with the actual population values at time *t* is (*s* denotes the initial condition)

(1) $$\begin{array}{c} X_{j}\left(t\right)=X_{j}\left(s\right)+\sum_{\alpha }\delta_{j}^{\alpha }n_{\alpha }\left(s,t\right). \end{array}$$

**Definition 1 (linear independency)**

One says that the populations are *linearly dependent* if there is a vector **u** ≠ 0 such that for all *t* ≥ *s* one has ∑_*j*_ *u*_*j*_ *X*_*j*_(*t*) = 0. Otherwise, one says that the populations are *linearly independent* and abbreviate it as LI.

In similar form, we say that the *populations have linearly dependent increments* if there is **u** ≠ 0 so that for all *t*′ > *t* ≥ *s* it holds that ∑_*j*_ *u*_*j*_(*X*_*j*_(*t*′) − *X*_*j*_(*t*)) = 0.

The populations are said to have *linearly independent increments* (abbreviated LII) if they do not have linearly dependent increments.

Along this work we will assume the following.

**Assumption 2**

The populations have *linearly independent increments*; that is, all involved populations are necessary to provide a complete description of the problem; none of them can be expressed as a linear combination of the remaining ones for all times.

**Lemma 3**

(a) If the populations have linearly independent increments, then the populations are linearly independent.

(b) The matrix *δ* has no nonzero vectors **u** such that ∑_*j*_ *δ*_*j*_^*α*^ *u*_*j*_ = 0 for all *α* if and only if the populations have linearly independent increments.

**Proof**

(a) The assertion is equivalent to the following: if the populations are linearly dependent, then they have linearly dependent increments, which is immediate after the definition of linearly dependent increments.

(b) Assume that for all *α* there exists **u** ≠ 0 such that ∑_*j*_ *u*_*j*_ *δ*_*j*_^*α*^ = 0; then

(2) $$\begin{array}{c} \sum_{j}u_{j}\left(X_{j}\left(t^{\prime }\right)-X_{j}\left(t\right)\right)=\sum_{j}u_{j}\left(\sum_{\alpha }\delta_{j}^{\alpha }n_{\alpha }\left(t^{\prime },t\right)\right)=0, \end{array}$$

and the populations have linearly dependent increments.

Conversely, assume that the populations have linearly dependent increments and select time intervals such that *n*_*β*_(*t*_*β*_′, *t*_*β*_) = 1 and *n*_*α*_(*t*_*β*_′, *t*_*β*_) = 0 for *α* ≠ *β*; then, we have that

(3) $$\begin{array}{c} \sum_{j}u_{j}\delta_{j}^{\alpha }=0,\;\forall \alpha . \end{array}$$

Statement (b) is the negative statement of these facts.

Strictly speaking the last step in the above proof takes for granted that there exist time intervals where only one event occurs. This is assured by the next assumption (third item). Also the initial conditions and the problem description should be such that all defined events are relevant for the dynamics (e.g., the event “contagion” is irrelevant for an epidemic problem with zero infectives as initial condition).

### 2.1. Dynamical Equation—Basic Assumptions

Population numbers evolve in “jumps” given by the occurrence of each event (birth, death, etc.). In the sequel, we will use Latin indices *i*, *j*, *k*, and *m* for populations and Greek indices *α*, *β*, and *γ* for events.

The ultimate goal of stochastic population dynamics is to calculate the probabilities *P*(*n*_1_,…, *n*_*E*_, *s*, *t*) of having *n*_1_,…, *n*_*E*_ events in each class up to time *t*, given an initial condition {*X*_*j*_(*s*)}.

The stochastic dynamics we are interested in correspond to a *Density Dependent Markov Jump Process* [37, 38]. For a sufficiently small time interval *h*, we consider the following.

**Assumption 4**

For each event,

- event occurrences in disjoint time intervals are independent;
- the Chapman-Kolmogorov equation [28, 29] holds;
- *P*(*n*_*α*_ + 1, *t* + *h*, *t*) = *W*_*α*_(*X*, *t*)*h* + *o*(*h*);
- *P*(*n*_*α*_, *t* + *h*, *t*) = 1 − *W*_*α*_(*X*, *t*)*h* + *o*(*h*);
- *P*(*n*_*α*_(*h*) + *k*, *t* + *h*, *t*) = *o*(*h*), *k* ≥ 2.

Here *h* is the elapsed time since the time *t* and *n*_*α*_ are the events of type *α* accumulated up to *t*.

It goes without saying that probabilities *P*(⋯) are assumed to be differentiable functions of time. *W*_*α*_(*X*, *t*) denotes the probability rate for event *α*, which is assumed in the following lemmas to be a smooth function of the populations, not necessarily linear (yet). Two well-known general results describe the dynamical evolution under these assumptions.

**Lemma 5 (see Theorem 1 in [29])**

Under Assumptions 2 and 4, the waiting time to the next event is exponentially distributed.

**Theorem 6 (see [28] and Theorem 1 in [29])**

Under Assumptions 2 and 4, the dynamics of the process in the space of events obeys the Kolmogorov Forward Equation, namely;

(4) P˙({n1,…,nE},s,t)  =∑αP({…,nα−1,…},s,t)Wα(X({…,nα−1,…,t}))   −P({n1,…,nE},s,t)(∑αWα(X({n1,…,nE,t}))).

Proofs of these results are given in Appendix A.

### 2.2. Linear Rates

We will assume that the transition rates depend—in a linear fashion—on the populations only. To distinguish from general rates (denoted above by the letter *W*), we will use the letter *R* to denote linear transition rates. Hence,

(5) $$\begin{array}{c} R_{\alpha }=\sum_{k}r_{\alpha }^{k}X_{k}\left(t\right). \end{array}$$

Since rates are nonnegative, we have that the proportionality coefficient between event *α* and subpopulation *k* satisfies *r*_*α*_^*k*^ ≥ 0. These proportionality coefficients convey the environmental information and as such they may be time-dependent since the environment varies with time. Whenever possible, we do not write this time dependency explicitly to lighten the notation.

**Lemma 7**

For the case of linear rates, population space {*X*_*k*_ ≥ 0} is positively invariant under the time evolution given by (1) and the previous assumptions if and only if *δ*_*i*_^*α*^ ≥ −1 and *δ*_*i*_^*α*^ = −1 only if *R*_*α*_ = *r*_*α*_^*i*^ *X*_*i*_(*t*) (no sum).

**Proof**

The probability of occurrence of event *α* in a small time interval *h* is

(6) $$\begin{array}{c} hR_{\alpha }+o\left(h\right)=h\sum_{k}r_{\alpha }^{k}X_{k}\left(t\right)+o\left(h\right). \end{array}$$

After an occurrence, population *X*_*k*_ is modified to *X*_*k*_ + *δ*_*k*_^*α*^. Population space is positively invariant under time evolution if and only if the probability of occurrence of event *α* is zero for those population values ${\bar{X}}_{k}$ such that ${\bar{X}}_{k}+\delta_{k}^{\alpha }<0$ (we are just saying that events pushing the populations to negative values do not exist). Since this should hold for arbitrary *h*, we have that *R*_*α*_ = 0.

Assume that *δ*_*k*_^*α*^ ≤ −1. Then, *R*_*α*_ = 0 for $0\leq {\bar{X}}_{k}<-\delta_{k}^{\alpha }$. Letting ${\bar{X}}_{k}=0$ we realise that ∑_*j*≠*k*_ *r*_*α*_^*j*^ *X*_*j*_(*t*) = 0 regardless of the values of *X*_*j*_, and hence it holds that *r*_*α*_^*j*^ = 0 for *j* ≠ *k* and therefore *R*_*α*_ = *r*_*α*_^*k*^ *X*_*k*_ (no sum). This proves the second statement. If *δ*_*k*_^*α*^ < −1 we have that *R*_*α*_ = 0 also for ${\bar{X}}_{k}=1$. Hence *r*_*α*_^*k*^ = 0 as well; *R*_*α*_ ≡ 0 and the event *α* can be ignored since it does not influence the dynamics.

## 3. Kolmogorov Forward Equation

Let us recast the dynamical equation given by Theorem 6 in terms of the generating function [39] in event-space, defined as

(7) $$\begin{array}{c} \Psi \left(z_{1},\ldots ,z_{E},s,t\right)=\sum_{\left\{n_{\alpha }\right\}}\left(\prod_{\alpha }z_{\alpha }^{n_{\alpha }}\right)P\left(n_{1},\ldots ,n_{E},s,t\right). \end{array}$$

For the case of linear rates, we define

(8) $$\begin{array}{c} R_{\alpha }^{0}=\sum_{k}r_{\alpha }^{k}X_{k}\left(s\right),\\ F_{\alpha }^{\beta }=\sum_{k}r_{\alpha }^{k}\delta_{k}^{\beta }. \end{array}$$

As a consequence,

(9) $$\begin{array}{c} R_{\alpha }=R_{\alpha }^{0}+\sum_{\beta }F_{\alpha }^{\beta }n^{\beta }. \end{array}$$

Using Theorem 6 to rewrite ∂Ψ/∂*t* and using further *n*_*β*_Ψ = *z*_*β*_(∂Ψ/∂*z*_*β*_) (a matter of replacing in the definition of Ψ), we obtain

(10) $$\begin{array}{c} {\frac{\partial \Psi }{\partial t}|}_{s}=\sum_{\alpha }\left(z_{\alpha }-1\right)\left(R_{\alpha }^{0}\Psi +\sum_{\beta }F_{\alpha }^{\beta }z_{\beta }\partial \Psi /\partial z_{\beta }\right). \end{array}$$

Since the generating function reflects a probability distribution, we have the probability condition Ψ(1,…1, *s*, *t*) = 1. Further, the solutions of (10) can be uniquely translated into those given by Theorem 6 for corresponding initial conditions: *P*(*m*_1_,…, *m*_*E*_, *s*, *s*) = 1 corresponding to Ψ(*z*_1_,…, *z*_*E*_, *s*, *s*) = *z*_1_^*m*_1_^ *z*_2_^*m*_2_^ ⋯ *z*_*E*_^*m*_*E*_^, where the integers *m*_1_,…, *m*_*E*_ denote how many events of each class had already occurred at *t* = *s*. The *natural initial condition* becomes Ψ(*z*_1_,…, *z*_*E*_, *s*, *s*) = 1, corresponding to *P*(0,…, 0, *s*, *s*) = 1.

### 3.1. The Method of Characteristics Revisited

**Lemma 8**

Equation (10) with *R*_*α*_^0^ = 0 and with the initial condition Ψ(*z*_1_,…, *z*_*E*_, *s*, *s*) = Φ_0_(*z*_1_,…, *z*_*E*_) admits the solution

(11) $$\begin{array}{c} \Psi \left(z_{1},\ldots ,z_{E},s,t\right)=\Phi_{0}\left(\phi \left(z,t,t-s\right)\right), \end{array}$$

where *ϕ*(*z*, *t*, *τ*) is the flow of the following ODE with initial condition *x*(0) = *z*, *s*(0) = *t*:

(12) $$\begin{array}{c} \frac{dx_{\beta }}{d\tau }=\sum_{\alpha }F_{\alpha }^{\beta }\left(x_{\alpha }-1\right)x_{\beta }\equiv b^{\beta }\left(x,s\right),\\ \frac{ds}{d\tau }=-1. \end{array}$$

**Proof**

It is convenient to rename *s* as *s* = *x*_*E*+1_, introduce *b* ^*E*+1^ = −1, and recall that *b* ^*β*^ depends only on the extended variable array *x*. However, when necessary, we will write explicitly *x*(0) as (*z*, *t*). We have that

(13) ∂Ψ∂t|s=∑βE+1∂Φ0∂xβ|ϕ(x,t−s)dϕβ(z,t,t−s)dt=∑βE+1∂Φ0∂xβ|ϕ(x,t−s)   ×(bβ(ϕ(z,t,t−s))+∂ϕβ(z,u,t−s)∂u|u=t).

Further, consider the monodromy matrix [40]

(14) $$\begin{array}{c} M_{\gamma }^{\beta }=\frac{\partial \phi^{\beta }\left(x,\tau \right)}{\partial x_{\gamma }}. \end{array}$$

*M* transforms the initial velocity into the final velocity; hence

(15) $$\begin{array}{c} \sum_{\gamma }M_{\gamma }^{\beta }b^{\gamma }\left(z,t\right)=b^{\beta }\left(\phi \left(z,t,\tau \right)\right). \end{array}$$

Thus, we get

(16) ∂Ψ∂t|s =∑βE+1∂Φ0∂xβ|ϕ(x,t−s)(∑γE+1Mγβbγ(z,t)+∂ϕβ(z,u,t−s)∂u|u=t) =∑βE+1∂Φ0∂xβ|ϕ(x,t−s)   ×(∑γEMγβbγ(z,t)−ME+1β+∂ϕβ(z,u,t−s)∂u|u=t) =∑γE∂Ψ∂zγbγ(z,t).

From the definition of monodromy matrix above it follows that

(17) $$\begin{array}{c} -M_{E+1}^{\beta }+{\frac{\partial \phi^{\beta }\left(z,u,t-s\right)}{\partial u}|}_{u=t}=0, \end{array}$$

and by (11) and the chain rule,

(18) ∑β∂Φ0∂xβ|x=ϕ(x,t−s)Mγβ=∑β∂Φ0∂xβ|x=ϕ(x,t−s)∂ϕβ(z,t,t−s)∂zγ=∂Ψ∂zγ.

**Lemma 9**

Equation (10) with *R*_*α*_^0^ ≠ 0 and with the initial condition Ψ(*z*_1_,…, *z*_*E*_, *s*, *s*) = Φ_0_(*z*_1_,…, *z*_*E*_) admits the solution

(19) $$\begin{array}{c} \Psi \left(z_{1},\ldots ,z_{E},s,t\right)=\Phi_{0}\left(\phi \left(z,t,t-s\right)\right)\Phi_{1}\left(z_{1},\ldots ,z_{E},s,t\right), \end{array}$$

where Φ_0_(*ϕ*(*z*, *t*, *t* − *s*)) is a solution of the homogeneous problem with *R*_*α*_^0^ = 0 and

(20) Φ1(z1,…,zE,s,t) =exp⁡∫st(∑αRα0(τ)(ϕα(z,t,t−τ)−1)dτ).

**Proof**

The proof is nothing more than an application of the method of the variation of the constants. The details are as follows.

Introducing the proposed solution Ψ into (10) and using Lemma 8 to eliminate Φ_0_ we obtain that Φ_1_ satisfies (10) with initial condition Φ_1_(*z*, *s*, *s*) = 1. Computing directly from (20) we have that

(21) ∂Φ1∂t|s =∑αRα0(ϕα(z,t,0)−1)Φ1+∑β∂Φ1∂ϕβ∂ϕβ(z,t,t−τ)∂t =∑αRα0(zα−1)Φ1  +∑β∂Φ1∂ϕβ(bβ(ϕ(z,t,t−τ))+∂ϕβ(z,u,t−τ)∂u|u=t) =∑αRα0(zα−1)Φ1+∑β∂Φ1∂ϕβ∑γMγβbγ(z,t) =∑αRα0(zα−1)Φ1+∑β∂Φ1∂ϕβ∑γ∂ϕβ∂zγbγ(z,t) =∑αRα0(zα−1)Φ1+∑γ∂Φ1∂zγbγ(z,t),

after retracing our steps from the previous Lemma.

The result presented in Lemma 9 was constructed using the method of variation of constants, which provides an alternative (constructive) proof.

### 3.2. Basic Properties of the Solution

**Definition 10**

A nonzero vector **v** such that ∑_*α*_ *δ*_*j*_^*α*^ *v*_*α*_ = 0, for *j* = 1,…, *N*, is called a *structural zero* of *F*. Other zero eigenvalues of *F* are called *nonstructural*.

**Lemma 11**

(a) There are structural zeroes if and only if *E* > *N*. (b) The only structurally stable zero eigenvalues of the matrix *F* are the structural zeroes.

**Proof**

(a) The “if” part is immediate since dim⁡(*ker*⁡(*δ*)) ≥ *E* − *N*. The “only if” part follows from the assumption of independent population increments (and therefore independent populations). For (b) put *F* = **r** *δ*. If *Fw* = 0 for *w* nonzero and *δw* = *p* ≠ 0, then **r** *p* = 0. This relation is not structurally stable since by adding *c* ≠ 0 arbitrarily small to all diagonal elements of **r** we can assure **r**′*p* ≠ 0 for this modified **r**′ and for any *p* ≠ 0.

**Lemma 12**

First integrals of motion for the characteristic equations. Let **v** be a structural zero of *F*, then

(22) $$\begin{array}{c} \prod_{\alpha }{\left(x_{\alpha }\left(t\right)\right)}^{v_{\alpha }}=C \end{array}$$

is a constant of motion for the characteristic equations (12).

**Proof**

Rewrite the first equation (12) as (indices renamed)

(23) $$\begin{array}{c} \frac{d}{dt}\ln x_{\alpha }=\sum_{\beta }F_{\beta }^{\alpha }\left(x_{\beta }-1\right). \end{array}$$

Then,

(24) $$\begin{array}{c} \sum_{\alpha }v_{\alpha }\frac{d}{dt}\ln x_{\alpha }=\sum_{\beta }\sum_{\alpha }\left(v_{\alpha }F_{\beta }^{\alpha }\right)\left(x_{\beta }-1\right)=0. \end{array}$$

Since ∑_*α*_(*v*_*α*_ *F*_*β*_^*α*^) = 0, the expression in the lemma is the exponential of the integral obtained from

(25) $$\begin{array}{c} \sum_{\alpha }v_{\alpha }\frac{d}{dt}\ln x_{\alpha }=0. \end{array}$$

Given relationship (1) between initial conditions, events, and populations, from the probability generating function in event space,

(26) $$\begin{array}{c} \Psi \left(z_{1},\ldots ,z_{E},s,t\right)=\sum_{\left\{n_{\alpha }\right\}}\left(\prod_{\alpha }z_{\alpha }^{n_{\alpha }}\right)P\left(n_{1}\cdots n_{E},s,t\right), \end{array}$$

a corresponding generating function in population space can be computed, namely,

(27) $$\begin{array}{c} \bar{\Psi }\left(y_{1},\ldots ,y_{N},s,t\right)=\sum_{\left\{X_{k}\right\}}\left(\prod_{k}y_{k}^{X_{k}}\right)P\left(X_{1},\ldots ,X_{N},s,t\right), \end{array}$$

letting {*n*_*α*_}, {*X*_*k*_} be shorthand for (*n*_1_ ⋯ *n*_*E*_), (*X*_1_,…, *X*_*N*_).

**Lemma 13 (projection lemma)**

Equation *z*_*α*_ = ∏_*k*=1_^*N*^ *y*_*k*_^*δ*_*k*_^*α*^^ realises the transformation from event space to population space. Moreover,

(28) Ψ¯(y1,…,yN,s,t)=(∏kykXk0)Ψ(z1,…,zE,s,t)=(∏kykXk0)Ψ(∏k=1Nykδk1,…,∏k=1NykδkE,s,t).

**Proof**

Taking logarithms on *z* and *y* it is clear that whenever there exist structural zeroes the transformation is noninvertible. In this sense we call it a *projection*. Concerning the statement, we have

(29) (∏kykXk0)Ψ(z1,…,zE,s,t) =(∏kykXk0)∑{nα}(∏α(∏k=1Nykδkα)nα)P({nα},s,t) =∑{nα}(∏k=1NykXk0+∑αδkαnα)P({nα},s,t).

The statement is proved using (1) and rearranging the sums as

(30) $$\begin{array}{c} P\left(X_{1},\ldots ,X_{N},s,t\right)={\sum_{\left\{n_{\alpha }\right\}}}^{\prime }P\left(\left\{n_{\alpha }\right\},s,t\right), \end{array}$$

where the prime denotes sum over {*n*_*α*_} such that *X*_*j*_^0^ + ∑_*α*_ *δ*_*j*_^*α*^ *n*_*α*_ = *X*_*j*_.

**Corollary 14**

The integrals of motion of Lemma 12 project under the projection Lemma to *C* = 1.

**Proof**

The above substitution operated on ∏*z*_*α*_^*v*_*α*_^ = *C* gives

(31) $$\begin{array}{c} C=\prod_{k=1}^{N}y_{k}^{\left(\sum_{\alpha }v_{\alpha }\delta_{k}^{\alpha }\right)}\equiv \prod_{k=1}^{N}y_{k}^{0}=1. \end{array}$$

### 3.3. Reduced Equation

**Assumption 15**

There exist integers *m*_*β*_, *β* = 1,…, *E* such that (cf. (9))

(32) $$\begin{array}{c} R_{\alpha }^{0}=\sum_{\beta }F_{\alpha }^{\beta }m_{\beta }. \end{array}$$

This assumption amounts to considering that there exists a way to mimic the initial condition on the rates with the same matrix *F* involved in the time evolution.

**Theorem 16**

Under Assumptions 2, 4, and 15, equation (10) with *natural* initial condition Ψ(*z*_1_,…, *z*_*E*_, *s*, *s*, ) = Φ_0_(*z*_1_,…, *z*_*E*_) = 1 has the solution

(33) $$\begin{array}{c} \Psi \left(z_{1},\ldots ,z_{E},s,t\right)=\prod_{\beta }f_{\beta }^{m_{\beta }}, \end{array}$$

where *z*_*β*_ *f*_*β*_(*z*, *s*, *t*) = *ϕ* ^*β*^(*z*, *t*, *t* − *s*) in terms of the solutions of (12) of Lemma 8. Hence, *f*(*z*, *s*, *t*) satisfies (after eliminating the auxiliary time *τ*)

(34) dlog⁡(fβ(z,s,t))ds =−∑αFαβ(s)(zαfα(z,s,t)−1), fβ(z,t,t)=1.

**Proof**

The differences between *f*( ) and *ϕ*( ) are (a) factoring out the initial condition *ϕ* ^*β*^(*z*, *t*, 0) = *z*_*β*_ and (b) rearranging the time arguments since the argument in *ϕ*( ) is natural to the ad hoc autonomous ODE, (12), while the argument in *f*( ) is natural to the (nonautonomous) PDE, (10).

The proof is just a matter of rewriting the previous equations under the present assumptions. Because of the natural initial condition, the desired solution to (10) is given by (20) in Lemma 9 which can be rewritten using Assumption 15, as (we use *z* as shorthand for *z*_1_,…, *z*_*E*_)

(35) Ψ(z,s,t)=Φ1(z,s,t)=∏β(exp⁡∫st∑αFαβ(τ)(ϕα(z,t,t−τ)−1)dτ)mβ.

Recalling that we defined *z*_*α*_ *f*_*α*_(*z*, *s*, *t*) = *ϕ*(*z*, *t*, *t* − *s*), the parenthesis can be recognised as the formal solution of (34) integrated from *t* to *s*.

**Corollary 17**

For **v** a structural zero of Lemma 12, the generating function is unmodified upon the substitution *m*_*α*_ → *m*_*α*_ + *kv*_*α*_ (*k* ∈ ℝ).

**Remark 18**

Having structural zeroes, it is a matter of choice to address the problem (a) using the variables *f*_*α*_ and imposing the additional constraints given by structural zeroes or (b) shifting to *population coordinates* where these constraints are automatically incorporated. Because of Assumption 2 and Lemma 12, the transformation mapping one description to the other corresponds to

(36) $$\begin{array}{c} f_{\alpha }=\prod_{j}w_{j}^{\delta_{j}^{\alpha }}. \end{array}$$

However, option (b) proves to be more efficient when it comes to deal with approximate solutions (see Section 5).

## 4. Examples of Exact Solutions

### 4.1. Example I: Pure Death Process

In the case *N* = *E* = 1, *δ* = −1, we have *r* > 0, *F* = −*r*(*s*), and *m* = −*M* < 0 (*M* is the initial population value). Equation (34) reads *df*/*ds* = *fr*(*zf* − 1), to be integrated in [*t*, *s*], with *f*(*z*, *t*, *t*) = 1. Hence, for constant death rate *r* we obtain *f*(*z*, *s*, *t*) = [*z*+(1−*z*)*e* ^−*r*(*t*−*s*)^]^−1^ and

(37) $$\begin{array}{c} \Psi \left(z,s,t\right)={\left[e^{-r(t-s)}+z\left(1-e^{-r(t-s)}\right)\right]}^{M}. \end{array}$$

### 4.2. Example II: Linear Birth and Death Process

We have *N* = 1, *E* = 2, and *δ*_1_^1^ = −*δ*_1_^2^ = 1, while *r*_1_ denotes the birth rate and *r*_2_ denotes the death rate. *X*_0_ = *m*_1_ − *m*_2_ is the initial population. There exists one structural zero, with vector **v** = (1,1)^*T*^, while the corresponding constant of motion results in the relations *z*_1_ *z*_2_ = *C* and *f*_1_ *f*_2_ = 1. The differential equation (34) for *f*_1_ (to be integrated in [*t*, *s*]) becomes

(38) $$\begin{array}{c} \frac{df_{1}}{ds}=\left(r_{1}z_{1}f_{1}^{2}-\left(r_{1}+r_{2}\right)f_{1}+\frac{Cr_{2}}{z_{1}}\right),\;f_{1}\left(z,t,t\right)=1, \end{array}$$

with solution (for *C* = 1 which will be the case after projection and assuming time-independent birth rates)

(39) $$\begin{array}{c} f_{1}\left(z,s,t\right)=\frac{1}{z_{1}}\frac{\left(r_{2}-r_{1}z_{1}\right)W-r_{2}\left(1-z_{1}\right)}{\left(r_{2}-r_{1}z_{1}\right)W-r_{1}\left(1-z_{1}\right)}, \end{array}$$

where *W* = exp⁡(*r*_2_ − *r*_1_)(*t* − *s*). Finally, Ψ(*z*, *s*, *t*) = (*f*_1_(*z*,*s*,*t*))^*m*_1_−*m*_2_^ which after projecting in population space using Lemma 13 and Corollary 14 gives (cf. [41])

(40) $$\begin{array}{c} \bar{\Psi }\left(y,s,t\right)={\left[\frac{\left(r_{2}-r_{1}y\right)W-r_{2}\left(1-y\right)}{\left(r_{2}-r_{1}y\right)W-r_{1}\left(1-y\right)}\right]}^{m_{1}-m_{2}}. \end{array}$$

**Remark 19**

A pure death linear process corresponds to a binomial distribution, a pure birth process corresponds to a negative binomial, and a birth-death process corresponds to a combination of both as independent processes.

## 5. Consistent Approximations

When (34) of Theorem 16 cannot be solved exactly, we have to rely on approximate solutions. It is desirable to work with *consistent approximations*, namely, those where basic properties of the problem are fulfilled exactly, rather than “up to an error of size *ϵ*”. For example, since our solutions should be probabilities, we want the coefficients of Ψ(*z*_1_,…, *z*_*E*_, *t*) to be always nonnegative and sum up to one. Ideally, we want approximations to be constrained to satisfy Lemma 12 and to preserve positive invariance in population space (no jumps into negative populations). Whenever consistency is not automatic, it is mandatory to analyse the approximate solution before jumping into conclusions.

### 5.1. Poisson Approximation

Let us replace *f*_*β*_ ≡ 1 in the RHS of (34) (this amounts to propose that *f*_*β*_ is constant and equal to its initial condition). The solution of the modified equation will be a good approximation to the exact solution for sufficiently short times such that *f* is not significantly different from one.

For constant rates *r*_*α*_ the rhs of (34) does not depend on time. The approximate solution reads *f*_*α*_(*z*, *s*, *t*) = exp⁡((*t* − *s*)∑_*β*_ *F*_*β*_^*α*^(*z*_*β*_ − 1)) and

(41) $$\begin{array}{c} \Psi \left(z_{1},\ldots ,z_{E},s,t\right)=\prod_{\alpha }^{E}e^{m_{\alpha }(t-s)\sum_{\beta }^{}F_{\beta }^{\alpha }(z_{\beta }-1)}=e^{\left(t-s)\sum_{\beta }^{}R_{\beta }^{0}(z_{\beta }-1\right)}. \end{array}$$

In other words, the generating function is approximated as a product of individual Poisson generating functions.

This approximation has an error of *o*(*t*). Simple as it looks, this approach gives a good probability generating function that respects structural zeroes, but it does not guarantee positive invariance: a Poisson jump could throw the system into negative populations. However, the probability of such a jump is also *o*(*t*). Fortunately, there is a natural way to impose positive invariance and also a natural way to improve the error up to *o*(*t*)^3/2^ in the broader framework of general transition rates [6]. In this way, the approximation can be safely used, with error control, to address problems that resist exact computation one way or the other.

### 5.2. Consistent Multinomial Approximations

A systematic approach to obtain a chain of approximations of increasing accuracy to (34) can be devised via Picard iterations. Firstly, it is convenient to recast the problem in population coordinates (cf.  Remark 18), so that the structural zeroes are automatically taken into account, regardless of other properties of the adopted approximation. From *f*_*α*_ = ∏_*j*_ *w*_*j*_^*δ*_*j*_^*α*^^ we obtain ${\dot{f}}_{\alpha }/f_{\alpha }=\sum_{k}\delta_{k}^{\alpha }{\dot{w}}_{k}/w_{k}$ and (34) can be recast as

(42) ∑kδkα(w˙kwk+∑βrβk(zβ(∏jwjδjβ)−1))=0,wk(z,t,t)=1.

Because of the linear independence of population increments (and hence of populations), Lemma 3(b), the counterpart of (34) in population coordinates reads

(43) $$\begin{array}{c} {\dot{w}}_{k}=-w_{k}\sum_{\beta }r_{\beta }^{k}\left(z_{\beta }\left(\prod_{j}w_{j}^{\delta_{j}^{\beta }}\right)-1\right),\;w_{k}\left(z,t,t\right)=1. \end{array}$$

**Remark 20**

In the case of linearly *dependent* population increments, (34) still holds, while it is possible to elaborate a more general form in terms of the *w*_*j*_ instead of (43). This is, however, outside the scope of this paper.

Since ∑_*α*_ *δ*_*j*_^*α*^ *m*_*α*_ = *X*_*j*_(*s*), we obtain Ψ(*z*_1_,…, *z*_*E*_, *s*, *t*) = ∏_*j*_ *w*_*j*_^*X*_*j*_(*s*)^, which is, so far, an exact result. Let us produce some approximated values for the *w*_*k*_'s, and correspondingly for Ψ(*z*_1_,…, *z*_*E*_, *s*, *t*).

To avoid further notational complications, we will assume in the sequel that *r*_*β*_^*k*^ is time independent. Hence, *w*_*k*_ will depend only on the time difference *t* − *s*. Equation (43) can be recast as

(44) $$\begin{array}{c} {\dot{w}}_{k}=w_{k}\sum_{\beta }r_{\beta }^{k}\left(z_{\beta }\left(\prod_{j}w_{j}^{\delta_{j}^{\beta }}\right)-1\right),\;w_{k}\left(z,0\right)=1, \end{array}$$

now to be integrated along the interval [0, *t* − *s*]. We will even set *s* = 0 in what follows.

**Remark 21**

For the case of time-independent proportionality factors *r*_*β*_^*k*^, a corresponding modification can be introduced in (34), namely, overall change of sign, integration in [0, *t* − *s*], and setting *s* = 0 to reduce the notational burden.

Equations (44) satisfy the Picard-Lindelöf theorem since the RHS is a Lipschitz function. Then the Picard iteration scheme converges to the (unique) solution. Taking advantage of the initial condition, the *n*th order truncated version of the Picard iterations reads

(45) wk(0)(h)=1,wk(1)(h)=1+∫0hwk(0)(t)∑βrβk(zβ∏j(wj(0)(t))δjβ−1)dt=1+h∑βrβk(zβ−1),wk(n)(h)=1+∫0hdt[wk(n−1)(t)×∑βrβk(zβ∏j(wj(n−1)(t))δjβ−1)]n−1,

where [⋯]_*m*_ denote the Maclaurin polynomial of order *m* in *t*.

**Lemma 22**

For *t* ∈ [0, *h*], *h* > 0 being sufficiently small, and for each order of approximation *n* ≥ 0, *w*_*k*_^(*n*)^(*t*) in (44) is a polynomial generating function for *one* individual of the population; that is, it has the following properties:

- *w*_*k*_^(*n*)^(*t*) is a polynomial of degree *n* in *z*_1_,…, *z*_*E*_, where the coefficient of *z*_1_^*n*_1_^,…, *z*_*E*_^*n*_*E*_^ is *O*(*t* ^*p*^), with *p* = *n*_1_ + ⋯+*n*_*E*_;
- *w*_*k*_^(*n*)^(1,…, 1, *t*) = 1;
- Δ_*k*_^(*n*)^(*h*) = *w*_*k*_^(*n*)^(*h*) − *w*_*k*_^(*n*−1)^(*h*) = *O*(*h* ^*n*^);
- the coefficients of *w*_*k*_^(*n*)^(*t*) regarded as a polynomial in {*z*_*α*_} are nonnegative functions of time.

**Proof**

(a) The statement holds for *n* = 0 and *n* = 1. Assuming that it holds up to order *n* − 1, we have that [*w*_*k*_^(*n*−1)^(*t*)∏_*j*_(*w*_*j*_^(*n*−1)^(*t*))^*δ*_*j*_^*β*^^]_*n*−1_ is also a polynomial of degree *n* − 1 in *z*_1_,…, *z*_*E*_ with time-dependent coefficients of type *O*(*t* ^*p*^) since each power *p* of *t* carries along a power *p* in {*z*_*α*_} (as well as lower *z*-powers). A Picard iteration step gives

(46) wk(n)(t)=1+∑βrβkzβ∫0h[wk(n−1)(t)∏j(wj(n−1)(t))δjβ]n−1dt −(∑βrβk)∫0hwk(n−1)(t)dt.

The first sum contributes with one time integration and one *z*-power, which exactly preserves the polynomial structure and the order of the coefficients. The result is a polynomial of degree *n* in *z*_1_,…, *z*_*E*_, where the coefficients are polynomials of highest degree at most *n* in *h*, each one of lowest order *O*(*h* ^*p*^) (as in the statement). The second integral does not alter these properties since it generates again a polynomial of degree *n* − 1 in *z*_1_,…, *z*_*E*_, with time-dependent coefficients of order *O*(*h* ^*p*+1^) or higher, up to *h* ^*n*^ (they are just the coefficients of *w*_*k*_^(*n*−1)^(*t*) integrated once in time).

(b) Letting *z*_1_ = *z*_2_ = ⋯ = *z*_*E*_ = 1, Picard iteration gives trivially *w*_*k*_^(*n*)^(*t*) = 1 to all orders, again by induction.

(c) The statement holds for *n* = 1. Assuming it holds up to *n* − 1, we compute

(47) wk(n)(h)−wk(n−1)(h) =∑βrβkzβ  ×∫0h([(wk(n−2)(t)+Δk(n−1)(t))      ×∏j(wj(n−2)(t)+Δj(n−1)(t))δjβ]n−1     −[wk(n−2)(t)∏j(wj(n−2)(t))δjβ]n−2)dt  −(∑βrβk)∫0h(wk(n−1)(t)−wk(n−2)(t))dt.

Both expressions in the first integral have the same Maclaurin polynomial up to order (*n* − 2). Hence, the difference in the first integral contains only terms of order *O*(*t* ^*n*−1^). This is also the case for the second integral. After integration, we obtain Δ_*k*_^(*n*)^(*h*) = *O*(*h* ^*n*^).

(d) The statement holds for *n* = 0 and *n* = 1 and in general for the zero-order coefficient, because it is unity for *t* = 0, as a consequence of the initial condition. For the higher-order coefficients, we use induction again. If all *z*-coefficients in *w*_*k*_^(*n*−1)^(*t*) are nonnegative, then the same holds automatically for *w*_*k*_^(*n*)^(*h*) up to order *n* − 1, since the *O*(*h* ^*n*^) contribution adding to each inherited coefficient from the previous step does not alter its sign, for *h* sufficiently small. It remains to show that the coefficients corresponding to *p* = *n* are nonnegative. These contributions arise from the (*n* − 1)th order of

(48) $$\begin{array}{c} r_{\beta }^{k}z_{\beta }{\left[w_{k}^{\left(n-1\right)}(t)\prod_{j}{\left(w_{j}^{\left(n-1\right)}(t)\right)}^{\delta_{j}^{\beta }}\right]}_{n-1} \end{array}$$

after time integration. If all *δ*_*j*_^*β*^ are positive, then the expression above is a product of *z*-polynomials with nonnegative coefficients and the statement follows. Suppose that some *δ*_*j*_^*β*^ = −1. Then by Lemma 7, *r*_*β*_^*k*^ = 0 for *k* ≠ *j*. Hence,

(49) rβkzβwk(n−1)(t)∏j(wj(n−1)(t))δjβ ={0,j≠k,rβkzβ∏j≠k(wj(n−1)(t))δjβ,j=k.

However, *δ*_*j*_^*β*^ ≠ −1 for *j* ≠ *k*; that is, they are nonnegative, and hence the expression has only nonnegative coefficients.

#### 5.2.1. Basic Properties of the First- and Second-Order Approximations

Let us analyse the generating function Ψ(*z*_1_,…, *z*_*E*_, *t*) = ∏_*j*_ *w*_*j*_^*X*_*j*_(0)^. The simplest nontrivial applications of the approximation scheme are the first- and second-order approximations. Expanding explicitly up to second-order, we get

(50) wk(h)=1+h∑βrβk(zβ−1)+h22(∑βrβk(zβ−1)) ×(∑γrγk(zγ−1)) +h22∑βrβkzβ∑γFγβ(zγ−1).

We begin by noticing that the first-order approximation is obtained by neglecting all terms in *h* ^2^ in the above equation. Whenever *w*_*j*_ is linear in *z*, which is always the case for the first-order approximation, the resulting generating function Ψ corresponds to the product of (independent) multinomial distributions for the events affecting each subpopulation.

Let us now consider Ψ(*z*_1_,…, *z*_*E*_, *t*) computed with the second-order approximated *w*_*j*_'s to perform one simulation step of size *h*.

Counting powers of *z*_*γ*_ on each *w*_*k*_ we have (a) the probability of no events occurring in time-step *h*

(51) $$\begin{array}{c} P_{k}\left(h,0\right)=1-h\left(\sum_{\beta }r_{\beta }^{k}\right)+\frac{h^{2}}{2}{\left(\sum_{\beta }r_{\beta }^{k}\right)}^{2}, \end{array}$$

(b) the probability of only one (*β*) event occurring in time-step *h*

(52) $$\begin{array}{c} P_{k}\left(h,I,\beta \right)=r_{\beta }^{k}\left\{h-h^{2}\sum_{\alpha }r_{\alpha }^{k}-\frac{h^{2}}{2}\sum_{\alpha }F_{\alpha }^{\beta }\right\}, \end{array}$$

and (c) the probability of another event (*α*) occurring after the first one:

(53) $$\begin{array}{c} P_{k}\left(h,II,\beta ,\alpha \right)=\frac{h^{2}}{2}r_{\beta }^{k}\left(r_{\alpha }^{k}+F_{\alpha }^{\beta }\right). \end{array}$$

#### 5.2.2. Simulation Algorithm

A simulation step can be operated in the following way. Let

(54) ∑α(Pk(h,II,β,α))+Pk(h,I,β)=rβk{h−h22∑αrαk}=P¯k(h,I,β)

be the probability of one (*β*) event occurring alone or followed by a second event. It follows that

(55) $$\begin{array}{c} P_{k}\left(h,II,\beta ,\alpha \right)={\bar{P}}_{k}\left(h,I,\beta \right)\;\frac{h}{2}\frac{\left(r_{k}^{\alpha }+F_{\alpha }^{\beta }\right)}{\left(1-\left(h/2\right)\sum_{\alpha }^{}r_{\alpha }^{k}\right)}+o\left(h^{2}\right). \end{array}$$

The second factor above is the conditional probability *P*_*k*_(*h*, *α*/*β*) for the second event being *α* given that there was a first (*β*) event.

The algorithm proceeds in two steps. First a multinomial deviate is computed with probabilities $\left\{{\bar{P}}_{k}(h,I,\beta )\right\}$ (the probability of no event occurring is still *P*_*k*_(*h*, 0)). This gives the number *n*_*β*_ of occurrences of each event. In the second step, for each *n*_*β*_ we compute the deviates for the occurrence of a second event or no more events, with the multinomial given by the array of probabilities {*P*_*k*_(*h*, *α*/*β*)} defined above (the probability of no second event occurring is here 1 − (*h*/2)(∑_*α*_(*r*_*k*_^*α*^ + *F*_*α*_^*β*^)/(1 − (*h*/2)∑_*α*_ *r*_*α*_^*k*^))).

## 6. Implementation: Developmental Cascades without Mortality

In this and the following Sections we apply the tools developed up to now to the description of a subprocess in insect development, namely, the development of immature larvae, which is, in principle, a highly individual subprocess. We assume that the evolution of an insect from egg to pupa occurs via a sequence of *E* > 0 maturation stages. Biologists recognise some substages by inspection in the development of larvae (instars) and even more stages when observed by other methods [35]. The immature individual may die along the process or otherwise complete it and exit as pupa or adult. The actual value of apparent maturation stages *E* will depend on environmental and experimental conditions and will ultimately be specified when analying experimental data in Section 7.1.

Empirical evidence based on existing and new experimental results as well as biological insight produced by the present description will be the subject of an independent work [42]. We discuss however a couple of examples from the literature in Section 7.1.

The events for this process are maturation events promoting individuals from one subpopulation into to the next and death events for all subpopulations. In other words, each subpopulation represents a given (intermediate) level of development and maturation events correspond to progress in the development. Development at level *j* promotes one individual to level *j* + 1 and the event rate is linear in the *j*-subpopulation. Hence, in *R*_*α*_ = ∑_*j*_ *r*_*α*_^*k*^ *X*_*j*_, the matrix *r* is square and diagonal, for 0 ≤ *α* ≤ *E* − 1 (we have exactly *E* + 1 subpopulations, counted from 0 to *E*). All of the environmental influence is, at this point, incorporated through *r*.

Moreover, level *E* is the matured pupa and we may set *r*_*E*_ = 0 since at pupation stage the present mechanism ends and new processes take place. Subpopulation *E* is the exit point of the dynamics and we assume it to be determined by the stochastic evolution of the immature stages 0 ≤ *α* ≤ *E* − 1.

In the same spirit, the action of each event on the populations modifies just the actual population and the next one in the chain. Hence,

(56) $$\begin{array}{c} \delta_{j}^{\alpha }=\{\begin{array}{cc} -1, & \alpha =j,\\ +1, & \alpha =j-1. \end{array} \end{array}$$

Following Section 3, [*R*_*α*_^0^]^*T*^ = (*r*_0_ *M*, 0,…, 0) and

(57) $$\begin{array}{c} F_{\alpha }^{\beta }=\{\begin{array}{cc} -r_{\alpha }, & \beta =\alpha ,\\ +r_{\alpha }, & \beta =\alpha -1,\\ 0, & \text{otherwise}. \end{array} \end{array}$$

Finally, letting **m** ^*T*^ = −*M*(1,1,…, 1) we can write *R*_*α*_^0^ = ∑_*β*_ *F*_*α*_^*β*^ *m*_*β*_. In this framework the index pair (*j*, *α*) is highly interconnected and we can achieve a complete description using just one index. We use 0 ≤ *j* ≤ *E* − 1 throughout this section.

The procedure of Section 3 gives Ψ({*z*}, *t*) = ∏_*j*_(*f*_*j*_)^*m*_*j*_^ = ∏_*j*_(*f*_*j*_^−*M*^), while the dynamical system for *f*_*j*_ reads, recalling the recasting in Remark 21;

(58) f˙j=fj(−rj(zjfj−1)+rj+1(zj+1fj+1−1)),j=0,…,E−2,

and ${\dot{f}}_{E-1}=f_{E-1}\left(-r_{E-1}(z_{E-1}f_{E-1}-1)\right)$. The overall initial condition is *f*_*j*_(0) = 1. Let *θ*_*j*_ = log⁡(*f*_*j*_) and

(59) $$\begin{array}{c} \psi_{j}=\sum_{\beta =j}^{E-1}\theta_{\beta };\;\;W_{j}=\exp\left(-\psi_{j}\right). \end{array}$$

Note that *W*_*j*_ *f*_*j*_ = *W*_*j*_ · exp⁡(*θ*_*j*_) = *W*_*j*+1_. Rewriting the equations,

(60) θ˙j=−rj(zjexp⁡(θj)−1)+rj+1(zj+1exp⁡(θj+1)−1),j<E−1,ψ˙j=−rj(zjexp⁡(θj)−1)=θ˙j+ψ˙j+1; j<E−1,θ˙E−1=ψ˙E−1=−rE−1(zE−1exp⁡(θE−1)−1),

with initial condition *θ*_*j*_(0) = 0 for all *j*. With this notation the generating function reads Ψ({*z*}, *t*) = *W*_0_^*M*^, and we formulate hence an ODE for the *W*_*j*_'s:

(61) $$\begin{array}{c} {\dot{W}}_{j}+r_{j}W_{j}=r_{j}z_{j}W_{j+1},\;\;W_{j}\left(0\right)=1;\;j=0,\ldots ,E-2,\\ {\dot{W}}_{E-1}=r_{E-1}\left(z_{E-1}-W_{E-1}\right),\;\;W_{E-1}\left(0\right)=1. \end{array}$$

By defining the auxiliary quantity *W*_*E*_(*t*) = 1, the solution for the *W*_*j*_-differential equations can be written in recursive form:

(62) $$\begin{array}{c} W_{j}\left(t\right)=e^{-r_{j}t}+z_{j}r_{j}\int_{0}^{t}e^{r_{j}(s-t)}W_{j+1}\left(s\right)ds,\;j=0,\ldots ,E-1. \end{array}$$

In Appendix B we give the details of the explicit solution of this problem in terms of Laplace transforms. Finally, we obtain

(63) W0(t)=(∏m=0E−1zm)(1−∑k=0E−1exp⁡(−rkt)∏j=0,j≠kE−1rjrj−rk) +∑k=1E∏j=0E−kzjzE−krE−k∑m=0E−krmexp⁡(−rmt)∏l=0,l≠mE−krlrl−rm.

The solution looks more compact when all *r*_*k*_ are assumed to take the common value *r*:

(64) W0(t)=∏m=0E−1zm(E−1)!∫0rtexp⁡(−s)sE−1ds +∑k=1E∏m=0E−kzmzE−k(rt)E−kexp⁡(−rt)(E−k)!.

## 7. Developmental Cascades with Mortality

We add to the previous description *E* subpopulation specific death events, *Q*_*α*_ = *q*_*α*_ *X*_*α*_, where *α* = 0,…, *E* − 1. We do not consider death of the pupa as an event for the same reasons as before: the pupa subpopulation *X*_*E*_ leaves the present framework of study. We have actually event pairs for each subpopulation and hence the analysis can still be done with just one index *α* = 0,…, *E* − 1, since each event is population specific. The calculations get however more involved. It is practical to organise the events as follows: *Q*_0_, *R*_0_, *Q*_1_, *R*_1_,…, *Q*_*E*−1_, and *R*_*E*−1_. Then, ((*Q*, *R*)^0^)^*T*^ = (*q*_0_ *M*, *r*_0_ *M*, 0,…, 0). We have

(65) $$\begin{array}{c} \delta_{\alpha }^{2\alpha }=-1,\left(\text{death}\right),\;\alpha <E,\\ \begin{array}{c} \delta_{\alpha }^{2\alpha +1}=-1\\ \delta_{\alpha +1}^{2\alpha +1}=+1 \end{array}\},\left(\text{development}\right),\;\alpha <E.\\ F_{2\alpha }^{\beta }=\{\begin{array}{cc} -q_{\alpha }, & \beta =2\alpha ,\\ -q_{\alpha }, & \beta =2\alpha +1,\\ q_{\alpha }, & \beta =2\alpha -1, \end{array}\\ F_{2\alpha +1}^{\beta }=\{\begin{array}{cc} -r_{\alpha }, & \beta =2\alpha +1,\\ -r_{\alpha }, & \beta =2\alpha ,\\ r_{\alpha }, & \beta =2\alpha -1, \end{array}\;\;\alpha <E. \end{array}$$

Recall that the indices in *F* run from 0 to 2*E* − 1. It is also practical to order the variables *f*_*α*_ and the *z*_*α*_ in pairs (*g*, *f*) and (*z*, *y*) as (*g*_0_, *f*_0_,…, *g*_*E*−1_, *f*_*E*−1_) and (*z*_0_, *y*_0_,…, *z*_*E*−1_, *y*_*E*−1_). Also for notational convenience we will assume that there exist *q*_*E*_ = *r*_*E*_ = 0 when necessary. Defining

(66) $$\begin{array}{c} H_{\alpha }\left(g_{\alpha },f_{\alpha }\right)=q_{\alpha }\left(z_{\alpha }g_{\alpha }-1\right)+r_{\alpha }\left(y_{\alpha }f_{\alpha }-1\right), \end{array}$$

we have (recall again Remark 21 and note that *H*_*E*_ ≡ 0),

(67) g˙α=−Hα(gα,fα)gα,f˙α=(−Hα(gα,fα)+Hα+1(gα+1,fα+1))fα,α=0,…,E−2,g˙E−1gE−1=f˙E−1fE−1=−HE−1(gE−1,fE−1).

The overall initial condition is still *g*_*α*_(0) = 1 = *f*_*α*_(0).

Since there are more events than subpopulations, according to Definition 1 and Corollary 14, *F* has structural zeroes corresponding to constants of motion in the associated dynamical system. The zero eigenvectors of *F* are *V*_*α*_^*T*^ = (0,…, −1,1, 1,0,…), nonzero in positions 2*α*, 2*α* + 1, and 2*α* + 2, for *α* = 0,…, *E* − 1 (for the last one, recall that there is no position “2*E*"). Definitions similar to those used in the previous section will be useful as well; namely, *θ*_*α*_ = log⁡*g*_*α*_ and *ϕ* ^*α*^ = log⁡*f*_*α*_, changing *H*_*α*_ and the initial conditions correspondingly. Hence,

(68) $$\begin{array}{c} {\dot{\phi }}_{\alpha }={\dot{\theta }}_{\alpha }-{\dot{\theta }}_{\alpha +1}⟹\phi_{\alpha }=\theta_{\alpha }-\theta_{\alpha +1},\;\alpha =0,\ldots ,E-2,\\ {\dot{\theta }}_{\alpha }=-H_{\alpha }\left(\theta_{\alpha },\theta_{\alpha }-\theta_{\alpha +1}\right),\;\alpha =0,\ldots ,E-2,\\ {\dot{\phi }}_{E-1}={\dot{\theta }}_{E-1}=-H_{E-1}\left(\theta_{E-1},\theta_{E-1}\right). \end{array}$$

The structural zeroes and constants of motion allow solving all equations related to the *ϕ*-variables and only a chain of *θ*-equations is left, very much in the spirit of the previous section. **m** ^*T*^ = −*M*(1,0,…, 0,0) satisfies *R* ^0^ = *Fm*. Finally, Ψ({*z*, *y*}, *t*) = *g*_0_^−*M*^. We now compute *g*_0_(*t*).

Let *W*_*α*_ = *e* ^−*θ*_*α*_^ = *g*_*α*_^−1^, 0 ≤ *α* ≤ *E* − 1. Defining the auxiliary variable *W*_*E*_(*t*) = 1, the dynamical equations for *θ* in terms of *W* read

(69) $$\begin{array}{c} {\dot{W}}_{\alpha }+\left(q_{\alpha }+r_{\alpha }\right)W_{\alpha }=q_{\alpha }z_{\alpha }+r_{\alpha }y_{\alpha }W_{\alpha +1},\;W_{\alpha }\left(0\right)=1, \end{array}$$

with solution

(70) Wα(t)=e−(qα+rα)t +∫0te−(qα+rα)(t−s)(qαzα+rαyαWα+1(s))ds.

In Appendix B we give the details of the explicit solution of this problem in terms of Laplace transforms. Finally, we obtain

(71) W0(t)=∏m=0E−1rmymqm+rm−∑m=0E−1rmyme−(qm+rm)tqm+rm ×∏k=0,k≠mE−1rkykqk−qm+rk−rm +∑k=1E1[ry]E−k   ×[∏m=0E−krmymqm+rm[qz]E−k     +∑m=0E−k(1−[qz]E−kqm+rm)×rmyme−(qm+rm)t     ×∏j=0,j≠mE−krjyjqj−qm+rj−rm].

Again we have that Ψ({*z*, *y*}, *t*) = *W*_0_^*M*^. Specialising for the case where all *r* and all *q* coefficients are equal, the solution reads

(72) W0(t)=∏m=0E−1rym(E−1)!∫0te−(q+r)xxE−1dx +∑k=0E−1∏m=0krymrykk![tke−(q+r)t+qzk∫0te−(q+r)xxkdx].

### 7.1. Examples from the Literature

In [19], Section 3, the drug delivery process by oral ingestion of a medicine consisting of microspheres containing the active principle is discussed. The process is modeled considering that microspheres enter the stomach (*E* = 0) and either dissolve (passing subsequently to the circulatory system) or proceed to the next stage of the GI tract, namely, the duodenum. Both processes are assumed to obey linear transition rates. In the same way, the undissolved duodenal microspheres pass to the next intestinal stage—the jejunum—and later to the ilium. The remaining undissolved microspheres entering the colon are eliminated without further dissolution. This is a neat example of a cascade, where dissolution corresponds to mortality in (71), while maturation corresponds to progress through the different stages. Remaining particles exit the system upon arrival to the fifth and last stage (*E* = 4). Using the data listed in page 239 of [19] for the dissolution and passage coefficients, the results of that paper can be directly read from (71). The probabilities of staying or dissolving at a given stage, function of time, appear as coefficients of *W*_0_ for different powers of *z*_*k*_ and *y*_*k*_. As an example, we compute the probabilities *p*_*i*_(*t*) of permanence at a given stage (as a function of time), using the *λ*- and *μ*-notation from page  239 in [19] (corresponding in (71) to *r* and *q*, resp.). Define first *P*(*s*) = ∏_*m*=0_^*s*^ *λ*_*m*_, *s* = 0,1, 2; *Q*(*s*, *m*) = ∏_*k*=0,*k*≠*m*_^*s*^(*λ*_*k*_ − *λ*_*m*_ + *μ*_*k*_ − *μ*_*m*_), *s* = 1,2, 3, for each integer *m* ∈ [0, *s*] and finally *R*(*s*) = *λ*_*s*_ + *μ*_*s*_, *s* = 0,…, 3. With these ingredients the probabilities are *p*_0_(*t*) = exp⁡(−*tR*_0_), while for *i* = 1,2, 3 we have *p*_*i*_(*t*) = *P*(*i* − 1)∑_*m*=0_^*i*^(exp⁡(−*tR*_*m*_)/*Q*(*i*, *m*)).

## 8. Concluding Remarks

The description of stochastic population dynamics at event level has several advantages with respect to the description at population level. While the projection onto population space is straightforward, the description of the number of events carries biological information that can be lost in the projection. From a practical, mathematical, point of view, the description in terms of events is more regular and makes room for a general discussion, since the number of events always increases by one.

When individual populations' processes are considered, the event rates become linear and, in addition, if the result of the event is to decrease one (sub)population, then they can only be proportional to the decreased population.

The probability generating function for the number of events, {*n*_*β*_}, conditioned to an initial state described by population values {*X*_*i*_(0)}, has been obtained as a function of the solutions of a set of ODE known as the equations of the characteristics but presented in a form oriented towards numerical calculus rather than the usual presentation oriented towards geometrical methods.

We have introduced short-time approximations that, to the lowest order, are in coincidence with previously considered heuristic proposals. Our presentation is systematic and can be carried out up to any desired order. In particular, we have shown how the second-order approximation can be implemented.

The general solution represents independent individuals identically distributed within their (sub)population compartment.

Additionally, we believe that obtaining general solutions of the linear problem is a sound starting point when the more complicated approximations for nonlinear rates are considered.

Further, we have solved a stochastic individual developmental model, that is, a model that at the population level is described by rates that are linear in the (sub)populations. The model not only describes just the populations but also describes the events that change the populations.

## Appendices

### A. Proofs of Traditional Results

The original version of these proofs (in slightly different flavours) can be found on page 428-9 (equations (48) and (50)) in [28] and equations (29) and (30) on page 498 in [29].

#### A.1. Proof of Lemma 5

Proof We assume the population and number of occurred events up to time *t* to be known. Let *τ* = *t*_1_ − *t* ≥ 0 be sufficiently small. It is clear that, if no events occur during the time interval *τ*, then *X*(*s*) = *X*(*t*), *t* ≤ *s* ≤ *t* + *τ*. For 0 ≤ *h* ≤ *τ* the Chapman-Kolmogorov equation implies that

(A.1) $$\begin{array}{c} P\left(n=0,t+h\right)=P\left(n=0,t\right)\left(1-\sum_{\alpha }W_{\alpha }\left(X,t\right)h\right)+o\left(h\right). \end{array}$$

Note that since *X* is constant along the time interval in consideration, the rates *W*(*X*, *t*) are well-defined. Hence,

(A.2) P(n=0,t+h)−P(n=0,t)h=−P(n=0,t) ×(∑αWα(X,t))+o(h)h,

and finally

(A.3) lim⁡h→0P(n=0,t+h)−P(n=0,t)h =P˙(n=0,t)=−P(n=0,t)(∑αWα(X,t)).

Given the *natural initial condition P*(*n* = 0, *t*) = 1, the solution to this equation is

(A.4) $$\begin{array}{c} P\left(n=0,t+\tau \right)=e^{-\tau \sum_{\alpha }^{}W_{\alpha }(X,t)}. \end{array}$$

#### A.2. Proof of Theorem 6

Proof Again, the Chapman-Kolmogorov equation gives

(A.5) P({n1,…,nE},t+h) =∑αP({…,nα−1,…},t)Wα(X({…,nα−1,…,t}))h  +P({n1,…,nE},t)(1−∑αWα(X({n1,…,nE,t}))h)  +o(h).

Repeating the previous procedure, taking the limit *h* → 0, we finally obtain Kolmogorov Forward Equation:

(A.6) P˙({n1,…,nE},t) =∑αP({…,nα−1,…},t)Wα(X({…,nα−1,…,t}))  −P({n1,…,nE},t)(∑αWα(X({n1,…,nE,t}))).

### B. Solution via Laplace Transform

After multiplying (62) by the Heaviside function *H*(*t*), we realise that the recursion involves a convolution. Hence, it is practical to use Laplace transforms. Indeed, defining *G*_*k*_(*s*) = *ℒ*(*H*(*t*)*W*_*k*_(*t*)), the equation can be rewritten as

(B.1) $$\begin{array}{c} G_{k}\left(s\right)=\frac{1}{s+r_{k}}\left(1+z_{k}r_{k}G_{k+1}\left(s\right)\right),\;G_{E}\left(s\right)=\frac{1}{s};\;0\leq k<E, \end{array}$$

since convolution product becomes standard product after transforming. After some manipulation, the recursion is solved by induction:

(B.2) $$\begin{array}{c} G_{0}\left(s\right)=\frac{1}{s}\prod_{m=0}^{E-1}\left(\frac{z_{m}r_{m}}{s+r_{m}}\right)+\sum_{k=1}^{E}\left(\frac{1}{z_{E-k}r_{E-k}}\prod_{m=0}^{E-k}\left(\frac{z_{m}r_{m}}{s+r_{m}}\right)\right). \end{array}$$

Inverse Laplace transform leads us back to (63). Note as well that when all *r*_*k*_ are taken to be equal, the corresponding *G*_0_(*s*) still gives the Laplace transform of the desired solution, while the inverse transform involves integrals related to the Gamma function (see (64)).

The case with mortality is similar, although slightly more complicated. Equation (70) reads, after Laplace transform as above (0 ≤ *k* < *E*),

(B.3) $$\begin{array}{c} G_{k}\left(s\right)=\frac{1}{s+q_{k}+r_{k}}\left(1+\frac{q_{k}z_{k}}{s}+r_{k}y_{k}G_{k+1}\left(s\right)\right),\\ G_{E}\left(s\right)=\frac{1}{s}, \end{array}$$

with solution

(B.4) $$\begin{array}{c} G_{0}\left(s\right)=\frac{1}{s}\prod_{m=0}^{E-1}\frac{r_{m}y_{m}}{s+q_{m}+r_{m}}+\sum_{k=1}^{E}{\left[\frac{s+qz}{sry}\right]}_{E-k}\prod_{\;m=0}^{E-k}\frac{r_{m}y_{m}}{s+q_{m}+r_{m}}. \end{array}$$

Again, inverse Laplace transform leads to (71).
